# MUC1 is a receptor for the *Salmonella* SiiE adhesin that enables apical invasion into enterocytes

**DOI:** 10.1371/journal.ppat.1007566

**Published:** 2019-02-04

**Authors:** Xinyue Li, Nancy M. C. Bleumink-Pluym, Yvette M. C. A. Luijkx, Richard W. Wubbolts, Jos P. M. van Putten, Karin Strijbis

**Affiliations:** 1 Department of Infectious Diseases & Immunology, Utrecht University, Utrecht, The Netherlands; 2 Department of Chemical Biology and Drug Discovery, Utrecht Institute for Pharmaceutical Sciences and Bijvoet Center for Biomolecular Research, Utrecht University, Utrecht, The Netherlands; 3 Department of Biochemistry and Cell Biology, Utrecht University, Utrecht, The Netherlands; University of California Davis School of Medicine, UNITED STATES

## Abstract

The cellular invasion machinery of the enteric pathogen *Salmonella* consists of a type III secretion system (T3SS) with injectable virulence factors that induce uptake by macropinocytosis. *Salmonella* invasion at the apical surface of intestinal epithelial cells is inefficient, presumably because of a glycosylated barrier formed by transmembrane mucins that prevents T3SS contact with host cells. We observed that *Salmonella* is capable of apical invasion of intestinal epithelial cells that express the transmembrane mucin MUC1. Knockout of MUC1 in HT29-MTX cells or removal of MUC1 sialic acids by neuraminidase treatment reduced *Salmonella* apical invasion but did not affect lateral invasion that is not hampered by a defensive barrier. A *Salmonella* deletion strain lacking the SiiE giant adhesin was unable to invade intestinal epithelial cells through MUC1. SiiE-positive *Salmonella* closely associated with the MUC1 layer at the apical surface, but invaded *Salmonella* were negative for the adhesin. Our findings uncover that the transmembrane mucin MUC1 is required for *Salmonella* SiiE-mediated entry of enterocytes via the apical route.

## Author summary

The bacterial pathogen *Salmonella enterica* is one of the most common causes of human foodborne infection affecting millions of people worldwide each year. To establish infection, *Salmonella* needs to cross the mucus layer and invade intestinal epithelial cells from the apical surface. However, the apical surface of intestinal epithelial cells is covered with a defensive barrier of large glycosylated transmembrane mucins. These large proteins prevent contact between the *Salmonella* type III secretion needle and the host plasma membrane thereby preventing invasion. We show for the first time that MUC1, one of the intestinal apical transmembrane mucins, facilitates *Salmonella* invasion. The *Salmonella* giant adhesin SiiE is the adhesin responsible for engaging MUC1 and the interaction is mediated by glycans on MUC1. We propose that SiiE interacts with MUC1 in a zipper-like manner that involves repetitive domains in both proteins. Adhesin-receptor interactions are essential for bacterial infection of host cells and key factors in determining target tissues and host range of bacteria. The SiiE-MUC1 invasion pathway may explain tropism of different *Salmonella* strains and provide a novel target for infection intervention and prevention.

## Introduction

In the gastrointestinal tract, the luminal microbiota is separated from the underlying epithelial cells by a complex system collectively called the mucus layer. The mucus layer consists of soluble gel-forming mucins such as MUC2 and MUC5A that are secreted by Goblet cells, IgA antibodies, host defense peptides, and other anti-microbial components [1]. Another component of the mucus layer are transmembrane mucins, which are large glycoproteins that are expressed on the apical surface of enterocytes and Goblet cells. Transmembrane mucins expressed in the gastrointestinal tract include MUC1, MUC3A, MUC3B, MUC4, MUC12, MUC13, MUC15, MUC17, MUC20 and MUC21 [2]. Transmembrane mucins have a highly glycosylated extracellular domain with potential barrier function, a transmembrane domain and a cytoplasmic tail that links to signaling pathways [3].

MUC1 is the most extensively studied transmembrane mucin and is highly expressed at mucosal surfaces including the stomach and the intestinal tract [4,5]. The MUC1 extracellular domain forms a large filamentous structure with a variable numbers of tandem repeats (VNTR) domain that can protrude 200–500 nm from the plasma membrane [6,7]. The extracellular domain is highly O-glycosylated with complex sugars that frequently terminate with sialic acids or fucose [8]. The human and mouse MUC1 extracellular domains share less than 40% homology while the transmembrane domain and cytoplasmic tail are highly conserved [9].

MUC1 plays an important role in defense against invasive bacterial pathogens such as *Helicobacter pylori* and *Campylobacter jejuni*. *In vitro* experiments with *H*. *pylori* and a gastrointestinal cell line showed that the extracellular domain of MUC1 is released and acts as a decoy that prevents bacterial attachment to cells [10]. Overexpression of MUC1 in HeLa cells or HCT116 cells protects against *C*. *jejuni* Cytolethal Distending Toxin (CDT) and CDT-treated cells internalize MUC1 into cytoplasmic vesicles or into the nucleus [11]. Expression of MUC1 in HCT116 cells increased adherence of *C*. *jejuni*, but invasion was not affected by MUC1 expression. The authors showed that *C*. *jejuni* adheres to O-glycan H type 2 sugars that contain a terminal fucose group [11]. In *in vivo* infection experiments, Muc1 knockout mice showed increased susceptibility to *H*. *pylori* and *C*. *jejuni* with more severe epithelial damage [10–12], but did not display increased susceptibility to *Salmonella* Typhimurium infection [11]. In addition to bacterial pathogens, MUC1 (over)expression also reduced infection by adenoviruses and influenza A [13–15].

*Salmonella enterica* is a food-borne, motile and facultative gastrointestinal pathogen. The non-typhoidal *Salmonella* (NTS) strains, *S*. *enterica* subsp. *enterica* serovar Enteritidis (*S*. Enteritidis) and *S*. *enterica* subsp. *enterica* serovar Typhimurium (*S*. Typhimurium) can cause self-limiting gastroenteritis in a wide range of hosts [16]. Three main routes have been described for *Salmonella* mucosal invasion: entry through M cells, direct invasion of enterocytes, and uptake through dendritic cells [17]. *Salmonella* cellular invasion is mediated by a type III secretion system that injects virulence factors into host cells to induce uptake. This process is well-studied for *Salmonella* invasion of different types of epithelial cells [18]. During intestinal pathogenesis *Salmonella* encounters the apical surface of intestinal epithelial cells where invasion is less efficient due to a defensive barrier of glycosylated transmembrane mucins. The transmembrane mucins presumably prevent contact of the T3SS with the host plasma membrane. The *Salmonella* giant adhesin SiiE that is secreted by the TISS and encoded by Pathogenicity Island 4 (SPI4) has been shown to be a key factor in apical invasion of polarized epithelial cells [19,20]. A specific receptor for SiiE that enables *Salmonella* apical entry has not been identified. In this study, we investigate the role of transmembrane mucin MUC1 during *Salmonella* invasion into intestinal epithelial cells.

## Results

### *Salmonella* apical invasion of intestinal epithelial cells coincides with MUC1 expression

**Fig 1:**
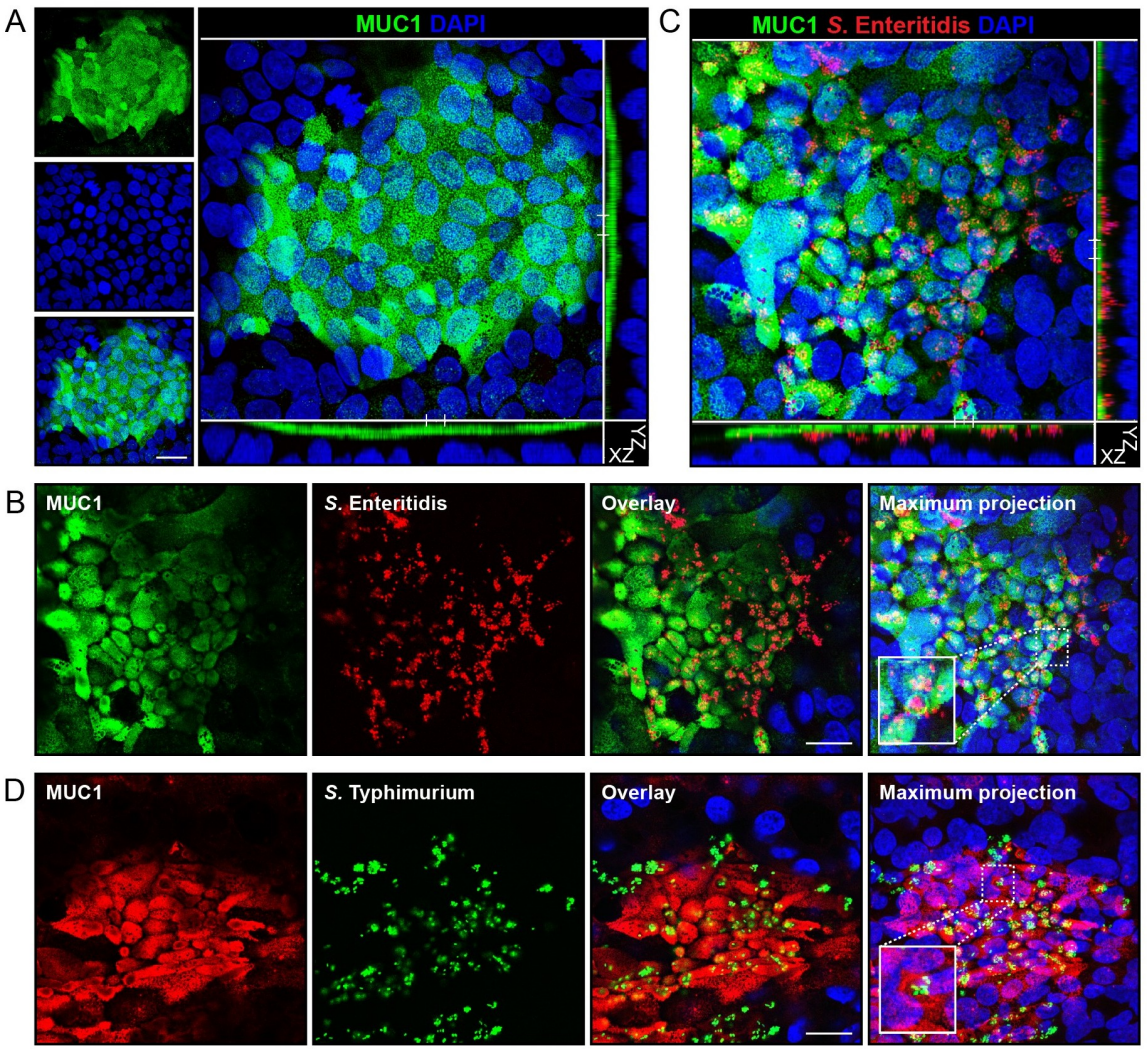
*Salmonella* invades intestinal HT29-MTX cells that express MUC1. (A) Immunofluorescence confocal microscopy imaging of confluent HT29-MTX cells with anti-MUC1 antibody 214D4 (green) and DAPI to stain the nuclei (blue). (B, C) Immunofluorescence confocal microscopy infection experiment with confluent HT29-MTX cells and *Salmonella enterica* Enteritidis (mCherry, red) at MOI 60 for 1 hour stained with anti-MUC1 214D4 antibody (green) and DAPI to stain the nuclei (blue). (D) Infection experiment with confluent HT29-MTX cells and *Salmonella enterica* Typhimurium (GFP, green) at MOI 60 for 1 hour stained with anti-MUC1 214D4 antibody (red) and DAPI (blue). White scale bars represent 20 μm.

Various human intestinal epithelial cell lines derived from colorectal carcinomas are used to study interactions with enteric pathogens, e.g. Caco-2, HT-29, HT29-MTX and HRT-18 cells. Previously it was shown that *Salmonella* apical adhesion and invasion is more effective in HT29-MTX cells compared to Caco-2 or HT-29 cells [21]. For this reason, we selected HT29-MTX cells to study the function of MUC1 during *Salmonella* invasion. To investigate MUC1 expression in HT29-MTX cells, we grew the cells for 5 days to form a confluent monolayer and performed immunofluorescence confocal microscopy with the 214D4 antibody directed against the MUC1 extracellular domain. MUC1 was expressed in a high percentage of HT29-MTX cells and localized to the apical side in an island-like pattern (Fig 1A).

To facilitate confocal microscopy of *Salmonella* invasion, we used *S*. Enteritidis containing a plasmid encoding red fluorescent mCherry. *S*. Enteritidis was grown till late logarithmic phase to achieve a high motile state and co-incubated with confluent HT29-MTX cells for 1 h. In this experimental setup, only the apical surface of the HT29-MTX cells is exposed to *Salmonella*. As revealed by confocal microscopy, *S*. Enteritidis invaded and formed clusters predominantly in MUC1 positive cells and did not invade adjacent MUC1-negative cells (Fig 1B). MUC1 seemed to localize to cup-like structures that contained large *Salmonella* clusters (Fig 1B). Multiple Z-stacks of the image were collected, and an orthogonal view clearly showed apical MUC1 with clusters of *Salmonella* located underneath (Fig 1C and S1A and S1B Fig). MUC1 did not colocalize with the intracellular *Salmonella*. Next, we investigated the contribution of the T3SS to apical invasion of HT29-MTX cells with a *Salmonella* Enteritidis CVI-1 wild type strain and its isogenic mutant *invG*, that is deficient in T3SS-mediated invasion. While the wild type strain did invade HT29-MTX cells at MUC1-positive loci, no adherent or invaded *invG* mutant bacteria could be detected (S1C Fig).

To determine if this phenomenon is restricted to *S*. Enteritidis, we also tested *S*. Typhimurium, another non-typhoidal *Salmonella*. We used *S*. Typhimurium containing a plasmid encoding GFP [22,23] and co- incubated the bacteria with confluent HT29-MTX cells. Comparable to *S*. Enteritidis, *S*. Typhimurium invaded and formed clusters in MUC1-expressing cells while MUC1-negative cells remained uninfected (Fig 1D). Again, MUC1-positive cup-like structures containing *Salmonella* clusters were observed. From these data we hypothesize that MUC1 positively contributes to the apical invasion of different *S*. *enterica* serovars into HT29-MTX cells.

### Generation of MUC1 knockout cells

**Fig 2:**
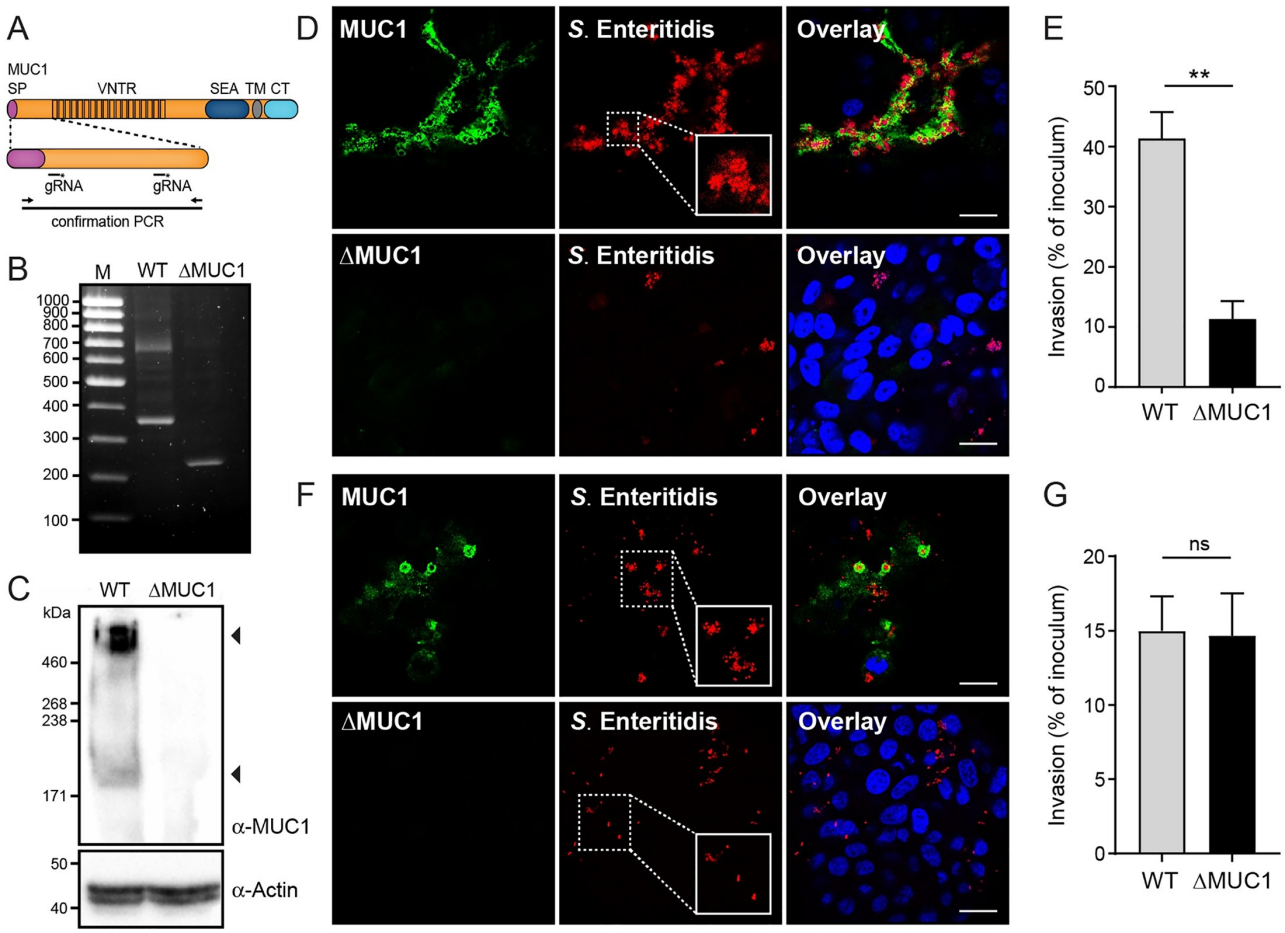
Knockout of MUC1 reduces *S*. Enteritidis apical invasion. (A) Schematic representation of the MUC1 coding region with different domains and the position of the guide RNAs (gRNAs) to generate a genetic deletion. SP, signal peptide; VNTR, variable numbers of tandem repeats; SEA, sea urchin sperm enterokinase agrin domain; TM, transmembrane domain; CT, cytoplasmic tail. The position of the deletion confirmation PCR primers is indicated. (B) Confirmation PCR of wild type HT29-MTX (WT) and HT29-MTX MUC1 knockout cells (ΔMUC1). (C) Western blot analysis of HT29-MTX wild type (WT) and MUC1 knockout cells (ΔMUC1) stained for MUC1 (214D4 antibody) or Actin. (D) Immunofluorescence confocal microscopy imaging showing apical invasion of confluent HT29-MTX wild type and ΔMUC1 cells after *S*. Enteritidis infection (1h) at MOI 60. MUC1 antibody 214D4 was used to stain MUC1 (green) and DAPI was used to stain nuclei (blue). (E) Quantification of *S*. Enteritidis invasion into confluent HT29-MTX wild type and ΔMUC1 cells. Confluent cells were incubated with *S*. Enteritidis for 1h at MOI 15 and subsequently treated with gentamicin (300 μg/ml). Cells were lysed and surviving intracellular bacteria were quantified by colony counts. Invasion is expressed as percentage of initial inoculum. Values are the mean ± SEM of three independent experiments performed in triplicate. (F) Immunofluorescence confocal microscopy imaging showing invasion of non-confluent HT29-MTX wild type and ΔMUC1 cells after *S*. Enteritidis infection as described under A. (G) Quantification of *S*. Enteritidis invasion into non-confluent HT29-MTX wild type and ΔMUC1 cells as described under E. Statistical analysis was performed by Student’s *t*-test using GraphPad Prism software. * *p*<0.05; ** *p*<0.01; ns, not significant. White scale bars represent 20 μm.

To determine the role of MUC1 in *S*. Enteritidis entry, we firstly designed a CRISPR/Cas9 genome editing strategy to knock out MUC1 in HT29-MTX cells (Fig 2A). Two guide RNAs (gRNAs) were selected in the 5’ end of the MUC1 gene that would generate a 130 bp deletion before the start of the tandem repeats. After transfection with the CRISPR-2xgRNA plasmid, cells were selected with puromycin and a positive single cell clone with the 130 bp deletion was identified (HT29-MTX-ΔMUC1; Fig 2B). Western blot analysis of wild type HT29-MTX cells revealed a reactive band of approximately 460 kDa that most likely represents fully glycosylated MUC1 and a band of about 170 kDa that presumably represents the unglycosylated precursor. Both bands were absent in the ΔMUC1 cells (Fig 2C).

### MUC1 facilitates *S*. Enteritidis apical entry of intestinal epithelial cells

Next, we performed *Salmonella* apical invasion experiments with the wild type and ΔMUC1 monolayers followed by confocal microscopy. *S*. Enteritidis invaded and formed clusters in the wild type cells as described above, but the bacteria barely invaded the ΔMUC1 cells (Fig 2D). To quantify this difference, we performed a quantitative invasion assay in which extracellular bacteria are killed with gentamicin and viable internalized bacteria are counted. This assay showed that approximately 41% of the introduced *S*. Enteritidis invaded the wild type cells, while about 11% of the bacteria invaded ΔMUC1 cells (p<0.01) (Fig 2E). The quantitative assay yielded a less pronounced effect compared to the microscopy. We hypothesize that this discrepancy is due to false positives in the quantification assay, a known problem associated with such assay that might be more pronounced during invasion at the apical surface. These data indicate that MUC1 does not form a barrier for *Salmonella* invasion but facilitates apical entry into HT29-MTX cells.

As *Salmonella* utilizes different strategies for apical or (baso)lateral invasion into intestinal epithelial cells, we also carried out invasion assays with 2-day grown non-confluent HT29-MTX WT and ΔMUC1 cells. These cells formed small islands with exposed lateral sides on the rim. MUC1 was only expressed in a limited number of cells in the center of the island. *S*. Enteritidis invaded both wild type and ΔMUC1 cell islands but showed a very different pattern. The large *S*. Enteritidis clusters described above were found exclusively in MUC1-expressing cells. A high percentage of ΔMUC1 cells was infected with S. Enteritidis through the lateral route, but only small red puncta of single bacteria could be observed (Fig 2F). In the apical invasion experiments with the confluent monolayers such small puncta were not observed. A quantitative invasion assay with non-confluent cells showed no difference in *S*. Enteritidis invasion between wild type and ΔMUC1 cells (Fig 2G). The formation of *Salmonella* clusters during apical invasion was previously described and only occurs in areas of membrane ruffle formation and macropinocytosis [24]. Our data suggest that MUC1 is associated with this apical process and that lateral invasion of *Salmonella* that is independent of MUC1 results in a different outcome. Collectively, these data demonstrate that MUC1 is an essential component for apical invasion of *S*. Enteritidis into confluent HT29-MTX cells but is not required for lateral entry of *S*. Enteritidis into non-polarized cells. Apical invasion through MUC1 appears to be very efficient and results in uptake of large clusters of *Salmonella* while individual bacteria internalize during lateral invasion.

### *Salmonella* invades different intestinal epithelial cell lines that express MUC1

**Fig 3:**
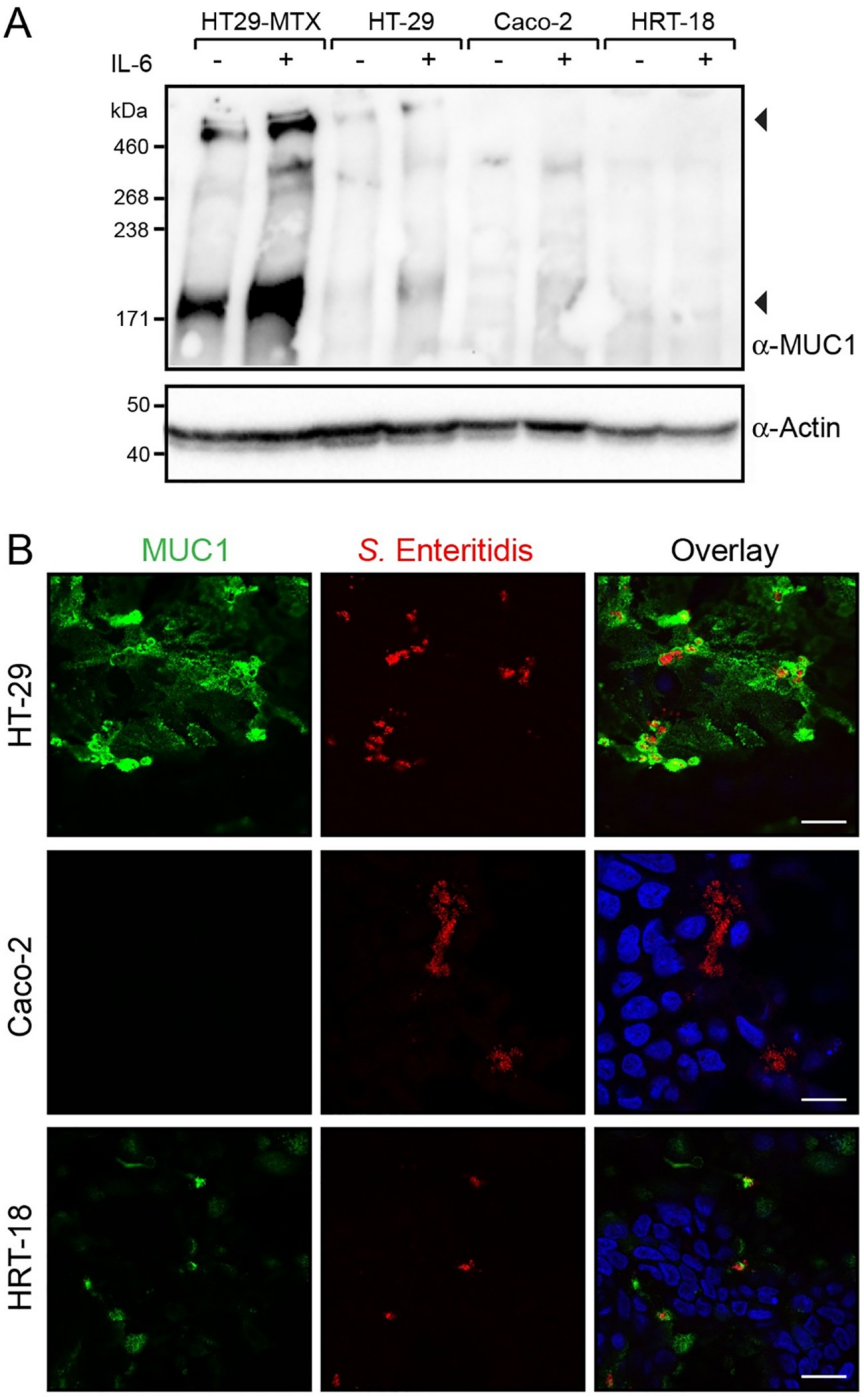
MUC1 expression and *Salmonella* invasion in different intestinal epithelial cell lines. (A) Western blot analysis of MUC1 expression levels in 5-day grown early-confluent intestinal epithelial cell lines incubated with or without the pro-inflammatory cytokine IL-6 at 100 ng/ml for 24h. (B) Immunofluorescence confocal microscopy imaging of 5-day grown early-confluent intestinal epithelial cell lines HT29-MTX, HT-29, Caco-2 and HRT-18 cells incubated with *S*. Enteritidis (mCherry, red) at MOI 60 for 1h stained with anti-MUC1 214D4 antibody (green) and DAPI (blue). White scale bars represent 20 μm.

To further substantiate the MUC1 contribution to *Salmonella* invasion, we performed apical invasion assays with early confluent monolayers of HT-29, Caco-2 and HRT-18 intestinal epithelial cell lines. Confocal microscopy showed that, under these conditions, HT-29, Caco-2 and HRT-18 cells express relatively low levels of MUC1 compared to HT29-MTX cells. Immunoblot analysis to determine MUC1 expression level in all cell lines indicated clear MUC1 bands in HT29-MTX and HT-29 cells, whereas MUC1 expression was below detection level in the other two cell lines after 5 days of culture (Fig 3A). Invasion assays showed that *Salmonella* did invade and form clusters in the MUC1-positive cells in the different cell lines although invasion levels seemed lower than detected for HT29-MTX cells (Fig 3B). MUC1 is reported to be upregulated under inflammatory conditions, for example after exposure to IL-6 [25,26]. Treatment of the early confluent monolayers with IL-6 strikingly increased MUC1 expression in HT29-MTX cells and HT-29 cells, but no difference was observed for Caco-2 and HRT-18 cells (Fig 3A). These results demonstrate variable MUC1 expression between cell lines and conditions and explain differences in apical invasion behavior of the pathogen between different intestinal epithelial cell lines.

### The interaction of *Salmonella* with MUC1 is mediated by sialic acids

**Fig 4:**
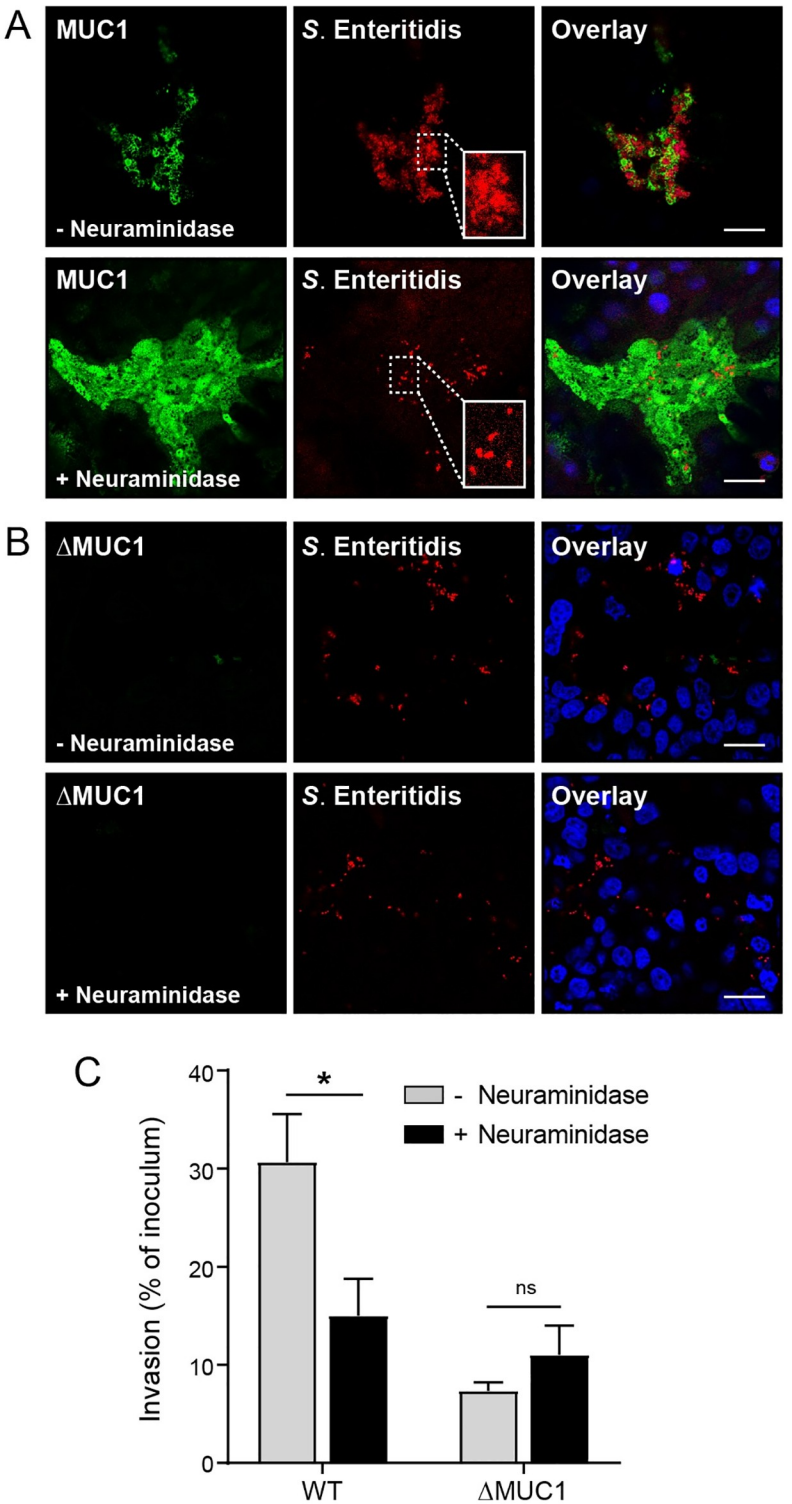
Deglycosylation with neuraminidase reduces *S*. Enteritidis apical invasion. Confluent HT29-MTX cells were treated with 250 mU/ml neuraminidase or DPBS for 2h followed by incubation with *S*. Enteritidis for 1 hour. (A) Immunofluorescence confocal microscopy images of confluent deglycosylated HT29-MTX cells incubated with S. Enteritidis (mCherry, red) stained for MUC1 (214D4, green) and nuclei (DAPI, blue). (B) Immunofluorescence confocal microscopy images of confluent deglycosylated ΔMUC1 cells incubated with S. Enteritidis (mCherry, red) stained for MUC1 (214D4, green) and nuclei (DAPI, blue). (C) Quantification of invaded intracellular *S*. Enteritidis in confluent HT29-MTX wild type and ΔMUC1 cells with and without neuraminidase treatment. After neuraminidase treatment, cells were incubated with *S*. Enteritidis for 1h at MOI 15 and subsequently treated with gentamicin (300 μg/ml). Cells were lysed and surviving intracellular bacteria were quantified by colony counts. Invasion is expressed as percentage of initial inoculum. Values are the mean ± SEM of three independent experiments performed in triplicate. Statistical analysis was performed by Student’s *t*-test using GraphPad Prism software. * *p*<0.05; ns, not significant. White scale bars represent 20 μm.

Alpha-2,3-linked sialic acids are essential for apical invasion of *S*. Typhimurium into MDCK cells [20]. The tandem repeats of the MUC1 extracellular domain are highly glycosylated with complex O-linked glycans on which sialic acids are frequently present [8]. To investigate if sialic acids on MUC1 are involved in apical invasion of *S*. Enteritidis into HT29-MTX cells, we removed α2,3-, α2,6-, and α2,8-linked sialic acids by neuraminidase treatment prior to the addition of *Salmonella*. Confocal microscopy showed that neuraminidase treatment of the HT29-MTX wild type cells resulted in a reduced *S*. Enteritidis invasion into wild type cells and a disappearance of *Salmonella* clusters (Fig 4A). Neuraminidase treatment did not affect *Salmonella* apical invasion into ΔMUC1 cells (Fig 4B). The quantitative invasion assay confirmed the significant reduction in *S*. Enteritidis invasion into wild type cells after neuraminidase treatment, whereas no significant difference was observed for ΔMUC1 cells (Fig 4B). Again, we did see that the quantitative assay yielded a less pronounced effect compared to the microscopy. Together these data clearly indicate that sialic acids on the extracellular domain of MUC1 are crucial for apical entry of *S*. Enteritidis into intestinal epithelial cells.

### The *Salmonella* adhesin SiiE is responsible for MUC1-mediated apical invasion

**Fig 5:**
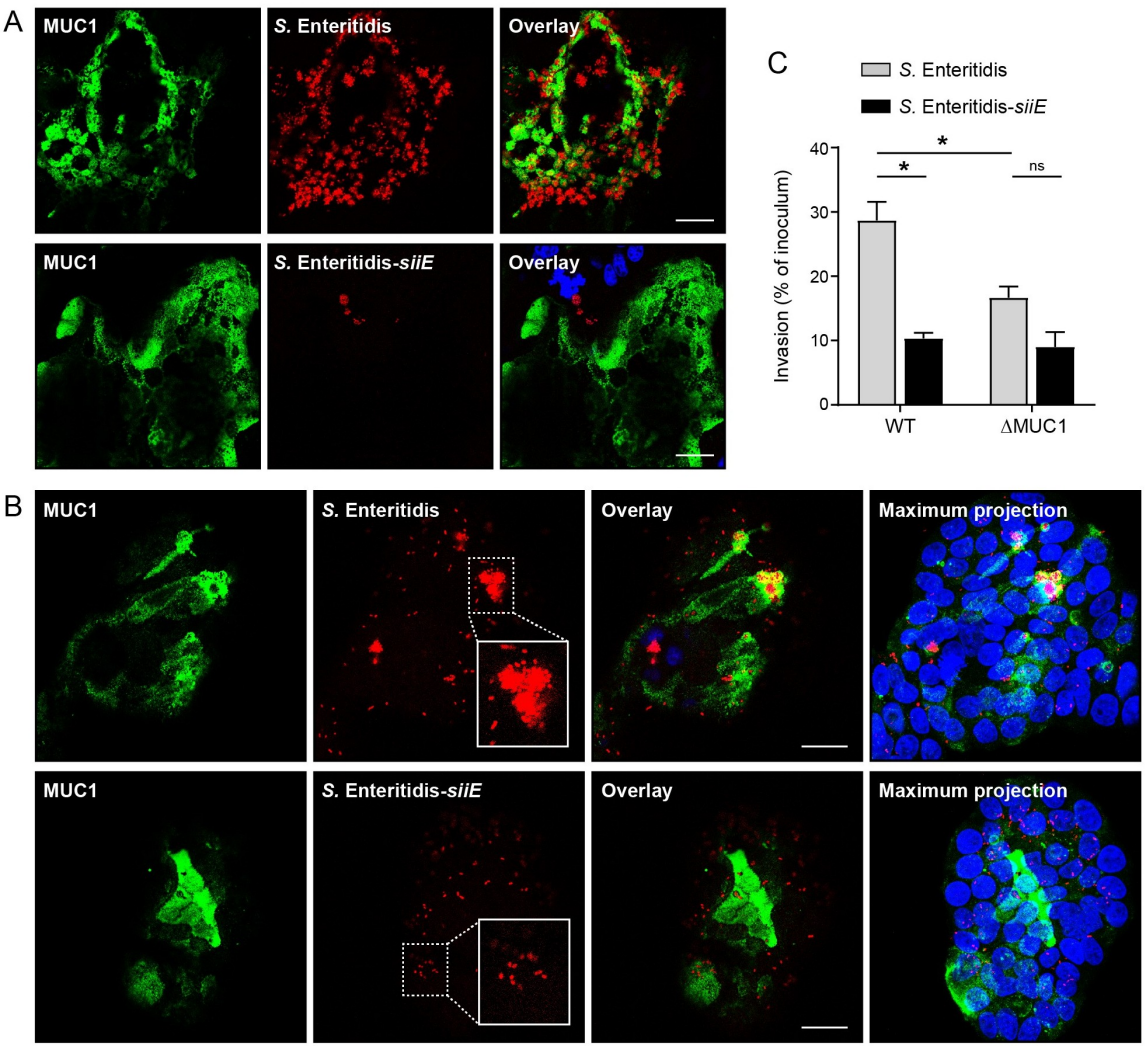
The *Salmonella* SiiE adhesin is responsible for MUC1-mediated invasion. (A) Immunofluorescence confocal microscopy imaging of confluent HT29-MTX cells infected with *S*. Enteritidis wild type and *siiE* knockout bacteria (mCherry, red) stained for MUC1 (214D4, green) and nuclei (DAPI, blue). (B) Immunofluorescence confocal microscopy imaging as above with non-confluent HT29-MTX cells. (C) Quantification of invaded intracellular *S*. Enteritidis wild type and *siiE* knockout bacteria. Confluent HT29-MTX cells were incubated with *S*. Enteritidis for 1h at MOI 15 and subsequently treated with gentamicin (300 μg/ml). Cells were lysed and surviving intracellular bacteria were quantified by colony counts. Invasion is expressed as percentage of initial inoculum. Values are the mean ± SEM of three independent experiments performed in triplicate. Statistical analysis was performed by Student’s *t*-test using GraphPad Prism software. * *p*<0.05; ns, not significant. White scale bars represent 20 μm.

*S*. *enterica* binding to receptors on epithelial cells is mediated by different adhesins. SiiE is a giant adhesin that mediates binding of *Salmonella* to the apical side of epithelial cells [19,27]. To determine if SiiE is the adhesin that contributes to binding of MUC1, we generated a *S*. Enteritidis *siiE* knockout strain. This strain demonstrated the same growth rate as the wild type strain. Wild type and *siiE* knockout bacteria were used in apical invasion assays with confluent HT29-MTX wild type cells. As shown by microscopy, the *siiE* knockout bacteria failed to invade HT29-MTX wild type cells (Fig 5A). To exclude the possibility that deletion of *siiE* affects *Salmonella* motility, we also performed a lateral invasion assay with non-confluent cells. Under these conditions, the wild type bacteria invaded from both the lateral side and the apical surface and the latter was associated with internalization of large bacterial clusters. The *siiE* knockout bacteria did invade the cells but no large bacterial clusters were detectable (Fig 5B). These data suggest that invasion through MUC1 was completely abolished in the *siiE* mutant bacteria. Quantification of *Salmonella* infection showed that invasion of the *siiE* mutant into HT29-MTX wild type cells was significantly reduced compared to *S*. Enteritidis wild type bacteria (p<0.05) (Fig 5C). Invasion of the *siiE* mutant into ΔMUC1 cells was low and not significantly altered (Fig 5C). Together, these data demonstrate that both MUC1 and SiiE are essential components for efficient apical entry of intestinal epithelial cells by *Salmonella*.

### The SiiE-MUC1 *Salmonella* invasion pathway enables apical invasion of intestinal epithelial cells

**Fig 6:**
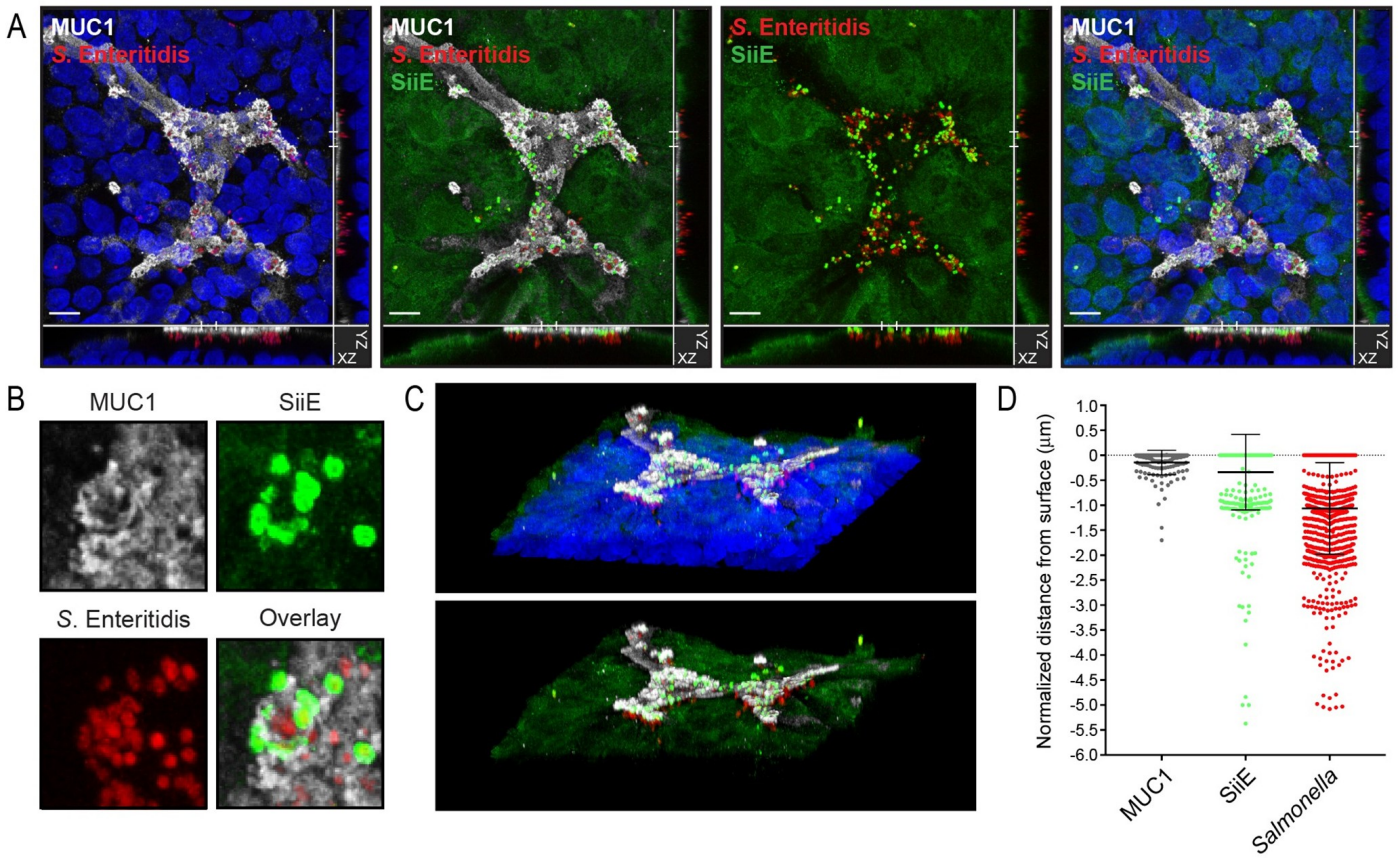
SiiE-positive *Salmonella* closely associate with MUC1 at the apical surface while invaded *Salmonella* are negative for SiiE (A) Immunofluorescence confocal microscopy imaging of confluent HT29-MTX cells infected with *S*. Enteritidis wild type bacteria (mCherry, red) stained for SiiE (polyclonal anti-SiiE, green), MUC1 (214D4, white) and nuclei (DAPI, blue). (B) Immunofluorescence confocal microscopy imaging detail showing localization of SiiE-positive bacteria (mCherry and green) on MUC1-positive cup-like structures (white). Bacteria inside the cup are negative for SiiE. (C) 3D projection of confocal microscopy image depicted in A. (D) Quantification and localization of SiiE-positive spots (green) and *Salmonella*-positive spots (red) relative to the MUC1-positive cellular surface using IMARIS software. White scale bars represent 20 μm.

**Fig 7:**
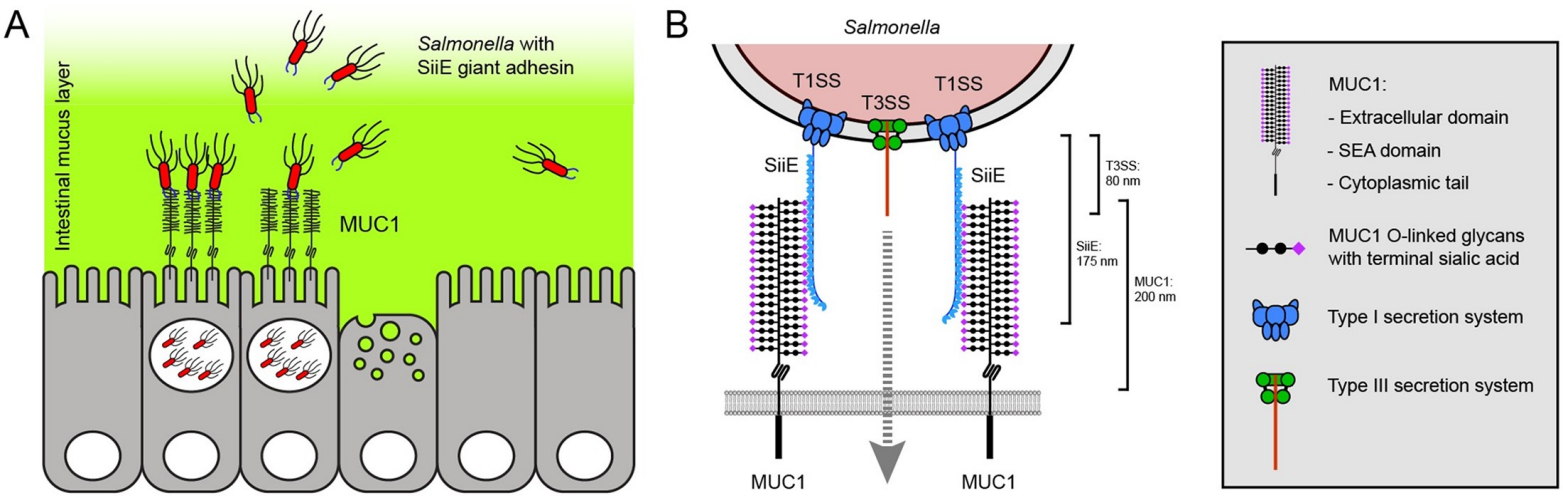
Schematic model showing the SiiE-MUC1 apical invasion pathway. (A) MUC1 is expressed on the apical surface of intestinal epithelial cells. *Salmonella* breaches the soluble mucus layer (green) and uses the giant adhesin SiiE to attach to MUC1 and invade the epithelial cells. After MUC1-SiiE-mediated invasion, large SiiE-negative *Salmonella* clusters are observed in the infected cells. (B) Hypothetical structural model of the SiiE-MUC1 interaction during *Salmonella* apical invasion of intestinal epithelial cells based on the molecular sizes of the involved players. The needle-like type III secretion system (T3SS) injectosome is essential for *Salmonella* invasion but is only 80 nm in length. The large transmembrane mucin MUC1 extends 200 nm from the cell surface and contains an extracellular domain with 42 tandem repeats that are highly decorated with O-linked glycans with terminal sialic acids. The giant adhesion SiiE is 175 nm in size and secreted through the type I secretion system (T1SS). SiiE contains 53 Blg repeats with sugar-binding capacity. Our data show that MUC1, sialic acids and SiiE are essential in mediating apical entry of *Salmonella*. We hypothesize that the SiiE-MUC1 interaction positions the *Salmonella* T3SS needle close enough to the host membrane to inject its virulence factors and induce uptake.

It has been reported previously that *Salmonella* secretes SiiE into the culture medium, but that the adhesin remains attached to the bacterial cell wall when contacting host cells [19]. In order to further investigate the function of SiiE during MUC1-mediated apical invasion we performed immunofluorescence confocal microscopy with an antibody directed against SiiE (kindly provided by Michael Hensel). As in our previous invasion experiments, *Salmonella* invaded MUC1-positive cells and the bacteria could be observed underneath the MUC1 surface. While SiiE-positive bacteria were closely associated with the MUC1 layer, *Salmonella* that had invaded further into the epithelial cells were negative for SiiE (Fig 6A, S2 Fig). SiiE-positive *Salmonella* associated closely with the MUC1-positive cups (Fig 6B). To visualize the process of SiiE-MUC1 mediated *Salmonella* invasion in more detail, we generated a 3D projection and an accompanying movie that includes a rotation (Fig 6C and S1 Movie). Using Imaris software, we determined the position of SiiE-positive and *Salmonella*-positive spots relative to the MUC1 surface layer in 5 independent Z-stacks. The SiiE-positive spots closely associated with the MUC1 layer, while the *Salmonella* spots localized close to the MUC1 layer and deeper into the cells (Fig 6D), conforming that invaded *Salmonella* are negative for SiiE. Together, our data show that glycosylated MUC1 acts as a receptor for *Salmonella* SiiE and enables SiiE-dependent apical invasion into the intestinal epithelium (Fig 7A).

## Discussion

Excellent work has shown that *Salmonella* T3SS enables cellular invasion, but bacterial entry into intestinal epithelial cells via the apical route appear less efficient due to a defensive barrier formed by transmembrane mucins. We demonstrate for the first time that *Salmonella* utilizes the transmembrane mucin MUC1 as a receptor to breach the apical barrier of intestinal epithelial cells. *Salmonella* efficiently invaded the apical surface of a monolayer of intestinal cells with high MUC1 expression whereas apical invasion into MUC1 knockout cells and cell lines that expressed low levels of MUC1 was severely reduced (Figs 2 and 3). Lateral invasion of non-confluent cells is not hampered by a defensive mucin layer and does not require MUC1 for entry (Fig 2). Enzymatic removal of sialic acids from intestinal epithelial cells reduced *Salmonella* invasion to MUC1 knockout levels, showing that sialic acids are instrumental for the MUC1-*Salmonella* interaction (Fig 4). MUC1-mediated invasion was abolished after the knock out of the *Salmonella* giant adhesin SiiE (Fig 5). SiiE-positive *Salmonella* closely associated with the MUC1 layer on the surface and invaded *Salmonella* were negative for SiiE (Fig 6). We propose that the SiiE-MUC1 invasion route is crucial to breach the epithelial mucin barrier and enable *Salmonella* apical invasion into MUC1-expressing enterocytes. A T3SS-1 mutant was unable to adhere or invade through MUC1, demonstrating that both the SiiE-MUC1 interaction and a functional T3SS are instrumental for successful *Salmonella* apical invasion.

Our data provide evidence that glycosylated MUC1 serves as a receptor for intestinal infection in several non-typhoid *Salmonella enterica* serovars. Different transmembrane mucins are expressed on the apical surface of intestinal epithelial cells and are generally considered to form a defensive barrier. The large filamentous glycoprotein MUC1 is widely expressed in mucosal tissues. Within the gastrointestinal tract, it is expressed in the stomach, duodenum and colon [2]. Previously, it was shown that MUC1 plays an important role in defense against the common enteric pathogens *H*. *pylori* and *C*. *jejuni* [10–12]. In contrast, our results showed that *Salmonella* exploits MUC1 for apical invasion into enterocytes. It is conceivable that other bacteria also make use of MUC1 for adhesion and invasion into host cells. For example, it has been reported that knockdown of MUC1 decreases invasion of *Staphylococcus aureus* into human corneal-limbal epithelial (HCLE) cells [28]. On the other hand, knockdown of the very long transmembrane mucin MUC16 increased invasion of *S*. *aureus*, suggesting that some mucin types have a barrier function for this bacterium. As the MUC protein family consists an array of transmembrane proteins with different extracellular and intracellular domains, it can be imagined that pathogens have evolved distinct strategies to breach the mucus layer and colonize the host.

The importance of MUC1 for *Salmonella* invasion via the apical route was further underpinned by the variable *Salmonella* entry in several commonly used intestinal epithelial cell lines. *Salmonella* apical infection efficiency clearly coincided with the level of MUC1 expression by these cell types. In non-polarized cells, *Salmonella* was capable of lateral invasion via a MUC1-independent route. These findings indicate that *Salmonella* can invade a single cell type through different mechanisms, dependent on the cell polarization status. We hypothesize that the SiiE-MUC1 interaction precedes contact of the T3SS with the host plasma membrane (see below).

*Salmonella* has been reported to exploit different mechanisms to invade the intestinal mucosa: (i) invasion through enterocytes, (ii) entry through M cells and (iii) uptake by mucosal dendritic cells [17]. It is conceivable that these seemingly redundant invasion mechanisms serve to colonize different regions of the gut and/or different species. Mouse and rabbit studies show that entry through M cells is a major route of *Salmonella* invasion [29,30]. *In vivo* studies in rabbits, calves and pigs have emphasized the importance of enterocyte invasion in addition to M cells [31–33]. To our knowledge, it is not known if M cells express MUC1. Moreover, MUC1 differs in composition and expression between species which theoretically may impact the preferred route of *Salmonella* invasion and tropism. For example, mouse Muc1 shares less than 40% homology with human MUC1 in the extracellular domain [9] and the pattern of protein glycosylation along the GI tract is opposite between mice and men. In humans, fucose decreases and sialic acid increases from ileum to colon [34], whereas in mice sialylated structures are the dominant terminal sugars in the small intestine and terminal fucose is more prevalent in the colon [35]. These differences may explain the need for different invasion routes but also make it complex to extrapolate results and to identify the predominant route of *Salmonella* invasion in different niches and species.

We identified the bacterial SiiE protein as the primary *Salmonella* adhesin involved in MUC1-mediated apical invasion. Genetic inactivation of the *siiE* gene reduced *Salmonella* entry from the apical side (Fig 5). The SiiE protein is expressed by most *S*. *enterica* serovars, but is a pseudogene in some highly host-adapted serotypes including *S*. Typhi [36]. The protein is encoded by *Salmonella* Pathogenicity Island 4 (SPI4) and secreted through a type I secretion system (T1SS) [19]. Association of SiiE with the bacterial surface is regulated by contact with polarized epithelial cells [37] and the protein interacts with MDCK cells in a lectin-like manner [20]. The sensitivity of the SiiE-mediated invasion for neuraminidase treatment (Fig 4) suggests that sialic acids on MUC1 are likely the primary target of SiiE. In colon, MUC1 is decorated with core 3 and core 4 glycostructures, with terminal fucoses or sialic acids [2,8] and this glycosylation pattern can be altered during *S*. Typhimurium infection [38]. In addition, MUC1 is upregulated under inflammatory conditions (Fig 3) [25,26] thereby increasing receptor availability. SiiE has been shown to contribute to *Salmonella* infections in mice. *S*. Enteritidis and *S*. Typhimurium strains lacking the SPI4 locus show a defect in intestinal colonization but are capable of establishing systemic infection [39].

A crucial aspect in the SiiE-MUC1 interaction is likely the structural architecture of both molecules. The SiiE protein is a giant adhesin (595 kDa) that contains an array of tandem repeats [37]. The extracellular domain of MUC1 also contains a (glycosylated) tandem repeat region. It can be imagined that the SiiE-MUC1 interaction involves sequential binding of the tandem repeats of both the adhesin and receptor. Such an interaction perfectly fits the need to breach the mucin barrier to correctly position the type III secretion system (T3SS) with its injectable virulence factors that are essential to initiate *Salmonella* invasion. It is important to consider that the MUC1 extracellular domain extends far from the plasma membrane and forms a barrier that is about 200–500 nm in size (6, 7). The T3SS injectosome, a needle-like machinery that is essential for successful invasion, is 80 nm in length [40]. Therefore, *Salmonella* needs additional virulence factors to breach the transmembrane mucin barrier and position the needle closer to the cell surface. SiiE has a rod-like structure and is 175 ± 5 nm in length [37]. Sequential interaction of the 53 highly conserved SiiE bacterial immunoglobulin (BIg) domains with the 42 highly glycosylated tandem repeats on MUC1 is likely a crucial first step to allow the T3SS system to initiate the invasion process. This scenario is supported by reports that increased deletion of the *Salmonella* SiiE Blg domains reduces bacterial invasion [41]. The mucus-binding protein MUB of *Lactobacillus reuteri* also contains multiple Blg-like repeats that bind intestinal mucus [42,43]. We hypothesize that SiiE interacts with MUC1 in a zipper-like manner (Fig 7B). Interaction of a single Blg domain with a single sialic acid is probably of low affinity, but multivalency of the adhesin-sugar interactions will provide strong avidity. We observed that during *Salmonella* invasion, MUC1 is localized to cup-like structures that contain large clusters of *Salmonella*. It remains to be investigated if MUC1 plays an active or a passive role in cup formation and bacterial uptake. We hypothesize that the SiiE-MUC1 interaction positions the T3SS needle close enough to the cell surface to inject its virulence factors and induce ruffle formation and *Salmonella* uptake. It is tempting to speculate that a cycle of SiiE release and reattachment of a novel molecule would enable the bacteria to move closer to the epithelial cell surface. Our hypothesis extends the previously proposed model for the function of SiiE in *Salmonella* apical invasion by providing evidence that MUC1 serves as the receptor for the SiiE adhesin.

In conclusion, we demonstrate for the first time that the transmembrane mucin MUC1 facilitates *Salmonella* apical entry into intestinal epithelial cells and serves as a receptor for the *Salmonella* giant adhesin SiiE. The exploitation of MUC1 by *Salmonella* underlines the importance of transmembrane mucins as targets in bacterial pathogenesis.

## Materials and methods

### Bacterial strains and growth conditions

*Salmonella enterica* serovar Enteritidis (*S*. Enteritidis) (strain 90-13-706, CVI, Lelystad) was transformed with plasmid pTVmCherry carrying the *mCherry* gene of *Discosoma* sp. optimized for bacterial expression (generously provided by J.M. Wells, Wageningen University). *S*. Enteritidis strains CVI-1 and CVI-1 *invG* were described previously [44]. Both strains were transformed with the pJET-mCherry plasmid (a kind gift from Mark Wösten, Utrecht University). *Salmonella enterica* serovar Typhimurium (*S*. Typhimurium) SL1344 carrying plasmid pMW85 expressing GFP from a PpagC promoter has been previously described [22,23]. *Salmonella* strains were routinely cultured at 37°C on LB agar plates containing kanamycin at 50 μg/ml or ampicillin at 100 μg/ml in 10 ml of LB broth while shaking (160 rpm). *Escherichia coli* DH5α used for cloning was grown on LB plates with the appropriate antibiotics.

### Construction of the *Salmonella* Enteritidis *siiE* knockout strain

The *S*. Enteritidis *siiE* knockout strain was constructed by deletion of the *siiE* gene using the lambda red homologous recombination system [45] combined with flanking regions similar to a strategy that was previously published [19]. The two regions flanking the *SiiE* gene were amplified with primers (KS290 5’- GTAGCATGCCAAAGGTATAGAACTCAAAAAGG-GTATCTGGA-3’ and KS291 5’- TACGGATCCACTCTCAAGGTGTATCTAATCGTTTAGT-3’ and KS292 5’-GTAGGATC-CCTCACCTTTGGGTGAGGGGGTTTAC-3 and KS293 5’-TACGTC-GACCTTCTGAGATAAAAATATTCCTGTTCTTCT-GTCC-3’) using the wild type *S*. Enteritidis genome DNA as a template and fused the respective sites of the spectinomycin adenylyltransferease-encoding gene [46] using overlap extension PCR. The final product was ligated into the pKO3 vector carrying a chloramphenicol resistance gene and electroporated into *S*. Enteritidis. After recovery in SOC medium (1h, 30°C), the bacteria were plated on LB plates with chloramphenicol and incubated at 43°C. The next day, single colonies were suspended into 1 ml of LB broth, serially diluted, and immediately plated on 8% sucrose-spectinomycin plates and incubated at 30°C. Gene replacement was verified by PCR with primers KS295 5’- GTTCATGGTCAGGGCGTTAT-3’ and 452 5’-GGCTG-CTCAAACTATACCAC-3’, 451 5’-AGGAGTATTTAAGCGAAGCAC-3’ and KS296 5’-GGAAATACGGCCAGAGACAAT-3’ and KS295/KS296.

### Mammalian cell lines and culture conditions

The human gastrointestinal epithelial cell lines HT29-MTX (a kind gift of Dr. Thécla Lesuffleur) [47], HT29-MTX-ΔMUC1 (derived from HT29-MTX wild type, this study), HT-29 (ATCC-HTB-38), Caco-2 (ATCC-HTB-37) and HRT-18 (ATCC-CCL-244) were routinely grown in 25 cm^2^ flasks in Dulbecco’s modified Eagle’s medium (DMEM) containing 10% fetal calf serum (FCS) at 37°C in 5% CO_2_. For *Salmonella* quantitative invasion assays, HT29-MTX wild type and ΔMUC1 cells were split into 12-well plates and grown for 5 days prior to addition of *Salmonella*. For microscopic cell imaging, cells were cultured on circular glass coverslips in 24-well plates.

### Generation of the HT29-MTX ΔMUC1 cell line using CRISPR/Cas9

To generate a ΔMUC1 cell line, we used the pCRISPR-hCas9-2xgRNA-Puro plasmid [48] that encodes Cas9 with 2 guide RNAs to generate a 130 bp deletion in the 5’ end of the MUC1 gene before the VNTR region. The pCRISPR plasmid was digested with *Sap*I and simultaneously dephosphorylated with alkaline phosphatase (FastAP; ThermoFisher). Guide RNA primer sets A (KS36 5’-ACCGGGTCATGCAAGCTCTACCCC-3’ and KS37 5’- AACGGGGTAGAGCTTGCATGACCC 3’) and B (KS131 5’-CCGGACATCCTGTCCCTGAGTGGG-3’ and KS132 5’- AAACCCACTCAGGGACAGGATGTC-3’) were phosphorylated with T4 polynucleotide kinase (ThermoFisher) at 37°C for 30 min and annealed by cooling down from 85°C to 25°C at 0.1°C/sec. Annealed primer sets were ligated into the *Sap*I-digested pCRISPR plasmid as confirmed by sequencing with primers KS46 5’- GTTCACGTAGTGCCAAGGTCG-3’ and KS47 5’-GAGTCAGTGAGCGAGGAAGC-3’, resulting in plasmid pCR11. Two-day grown HT29-MTX cells were trypsinized from a 25 cm^2^ flask and transfected in suspension with 2 μg of pCR11, pCRISPR-empty or no plasmid using Fugene (Promega) according to the manufacturer’s instructions. Cells were cultured in DMEM + 10% FCS for two days, after which 5 μg/ml puromycine (Life Technologies) was added to the medium to select for positively transfected cells. Cells were maintained in medium with puromycine until all negative control cells had died. Single cell cloning was performed by serial dilution and single cell clones were tested for the MUC1 deletion by PCR with primers KS133 5’-CAGTCCTCCTGGTATTATTTCTCTGGTG-3’ and KS134 5’- CAGGTGGCAGCTGAACCTGAAG-3’. The absence of MUC1 protein in the HT29 MTX-ΔMUC1 cell line was confirmed by immunoblot with mouse monoclonal antibody 214D4 (a kind gift from Dr. John Hilkens; CD227, Nordic MUBio) directed against the MUC1 tandem repeats.

### Confocal microscopy

Cells were grown on cover slips (8 mm diameter #1.5) in 24-well plates and for bacterial invasion studies, *Salmonella* was grown as described below and added at a multiplicity of infection (MOI) of 60 for 1 h. Then cells were washed twice with Dulbecco’s Phosphate Buffered Saline (DPBS, D8537, Sigma) and fixed with 4% cold paraformaldehyde in PBS (Affimetrix) for 30 min at room temperature. Cells were rinsed twice with DPBS before they were permeabilized in binding buffer (0.1% saponin, Sigma and 0.2% BSA, Sigma in DPBS) for 30 min. Next, coverslips were incubated with α-MUC1 antibody 214D4 at 1:150 dilution and/or α-SiiE rabbit polyclonal antiserum (1:200; a kind gift of Michael Hensel, University of Osnabrück) for 1h followed by 4 washing steps with binding buffer. The cover slips were incubated with Alexa Fluor-488/568-conjugated goat α-mouse IgG secondary antibodies (1:200; A11029, A11031; ThermoFisher), goat α-rabbit IgG secondary antibodies (1:200; A11034, A11036; ThermoFisher) and Alexa Fluor-647-conjugated donkey α-mouse IgG secondary antibody (1:200; 715-605-151; Jackson ImmunoResearch) and DAPI at 2 μg/ml (D21490, Invitrogen) for 1h. Coverslips were washed 3 times with DPBS, once with MilliQ, dried and embedded in Prolong diamond mounting solution (ThermoFisher) and allowed to harden. Images were collected on a Leica SPE-II confocal microscope using a 63x objective (NA 1.3, HCX PLANAPO oil) controlled by Leica LAS AF software with default settings to detect DAPI, Alexa488, Alexa568 and Alexa647. Axial series were collected with step sizes of 1 μm. Object centroid positions in Z were determined in IMARIS 8.2 (Bitplane, UK) on images that were deconvolved using the 3D automated deconvolution package in NIS elements (NIKON). *Salmonella* and SiiE segmented objects were created using the spot detection wizard and MUC1-labeled positions were isolated using the surface detection wizard in 5 image stacks. Centroid Z positions of the of the objects were extracted in the Vantage module and exported to Excel (Microsoft, Redmond USA) for plotting.

### Quantitative invasion assay

*Salmonella* overnight cultures (16 h) were diluted 1:30 in LB broth and grown for 3.5 h at 37°C till late logarithmic phase. After adjustment of the OD_600_ to 0.24, 1 ml of bacterial culture was centrifuged at 8000 rpm for 2 min. The bacterial pellet was resuspended in 1 ml DPBS, immediately 30 μl of bacterial suspension was added to cells in a 12-well plate in DMEM without FCS (MOI 15) and incubated for 1 h at 37°C. Cells were rinsed twice with DPBS, placed in DMEM without FCS containing 300 μg/mL of gentamicin, and incubated for 1 h at 37°C to kill extracellular bacteria. Cells were then washed twice with DPBS and lysed with lysis buffer (0.1% Triton X-100 in DPBS) for 5 min at 37°C. Serial dilutions were made and plated on LB agar plates containing 50 μg/mL kanamycin. The next day, colonies were counted.

### Western blot

Protein samples were prepared from cells cultured in 6-wells plates. The cells were washed once with DPBS and 200 μl of lysis buffer was added to each well to detach the cells. Then 100 μl of 3x Laemmli sample buffer was added to the lysate followed by boiling for 5 min at 100°C. For immunoblotting of large mucin proteins, a Boric acid-Tris system was developed. A 5% mucin gel (12.5% 40% Acryl/Bis acryl, Biorad 161–0144; 26% 1.5 M Tris pH 8.8, Invitrogen; 10% SDS, Invitrogen; 10% ammonium persulfate, Invitrogen; 0.1% TEMED, ThermoFisher) was made in a Mini Protean II chamber (Bio-rad) using 1.5 mm spacer plates. Boiled protein lysates were loaded onto the gel, and run in Boric acid-Tris buffer (192 mM Boric acid, Merck; 1 mM EDTA, Merck; 0.1% SDS, to pH 7.6 with Tris) at 25 mA for 1.5 h. Protein was transferred onto a nitrocellulose membrane using a wet transfer system with transfer buffer (25 mM Tris; 192 mM glycine, Merck; 20% methanol, Merck) for 3 h at 90 V at 4°C. Subsequently, the membranes were blocked with 5% BSA in TSMT (20 mM Tris; 150 mM NaCl, Merck; 1 mM CaCl_2_, Sigma; 2 mM MgCl_2_, Merck; adjusted to pH 7 with HCl; 0.1% Tween 20, Sigma) overnight at 4°C. The next day, the membrane was incubated with 214D4 antibody at a dilution of 1:150 in TSMT containing 1% BSA for 1 h at RT, washed 2 times with TSMT, 2 times with DPBS and incubated with α-mouse IgG secondary antibody (A2304, Sigma) diluted 1:8000 in TSMT with 1% BSA for 1 h at RT. For detection of actin, cell lysates were loaded onto a 10% SDS-PAGE gel, transferred to PVDF membranes and incubated with α-Actin antibody (1:5,000; bs-0061R, Bioss) and α-rabbit IgG (1: 10,000; A4914, Sigma). Blots were developed with the Clarity Western ECL kit (Bio-Rad) and imaged in a Gel-Doc system (Bio-Rad).

### Enzymatic removal of sialic acids

Desialylation of HT29-MTX and ΔMUC1 cells was achieved by incubating cells grown in a 24-well plate with 250 mU/mL neuraminidase (Sigma) in DPBS for 2 h at 37°C. Cells were then washed twice with DPBS and used in *Salmonella* invasion assays.

## Supporting information

S1 Fig (Accompanies Fig 1). (A) Single channel maximum projections with orthogonal view of the image depicted in Fig 1B and 1C. MUC1: green, *Salmonella*: red, nuclei: blue. (B) Montage of different Z planes of the image depicted in Fig 1B and 1C. MUC1: green, *Salmonella*: red, nuclei: blue. (C) Immunofluorescence confocal microscopy imaging of confluent HT29-MTX cells infected with *S*. Enteritidis CVI-1 wild type or *invG* knockout bacteria (mCherry, red) stained for MUC1 (214D4, green) and nuclei (DAPI, blue). White scale bars represent 20 μm.
(TIF)

S2 Fig (Accompanies Fig 6). Montage of different Z planes of the image depicted in Fig 6A. MUC1: white, SiiE: green, *Salmonella*: red, nuclei: blue.
(TIF)

S1 Movie 3D projection and rotation of confocal microscopy image depicted in Fig 6A. MUC1: white, SiiE: green, *Salmonella*: red, nuclei: blue.
(MOV)
