# Supplementary figures and images for: MUC1 is a receptor for the Salmonella SiiE adhesin that enables apical invasion into enterocytes
Source: PLoS Pathog. 2019 Feb 4;15(2):e1007566. doi: 10.1371/journal.ppat.1007566 (PMC6375660; doi:10.1371/journal.ppat.1007566)

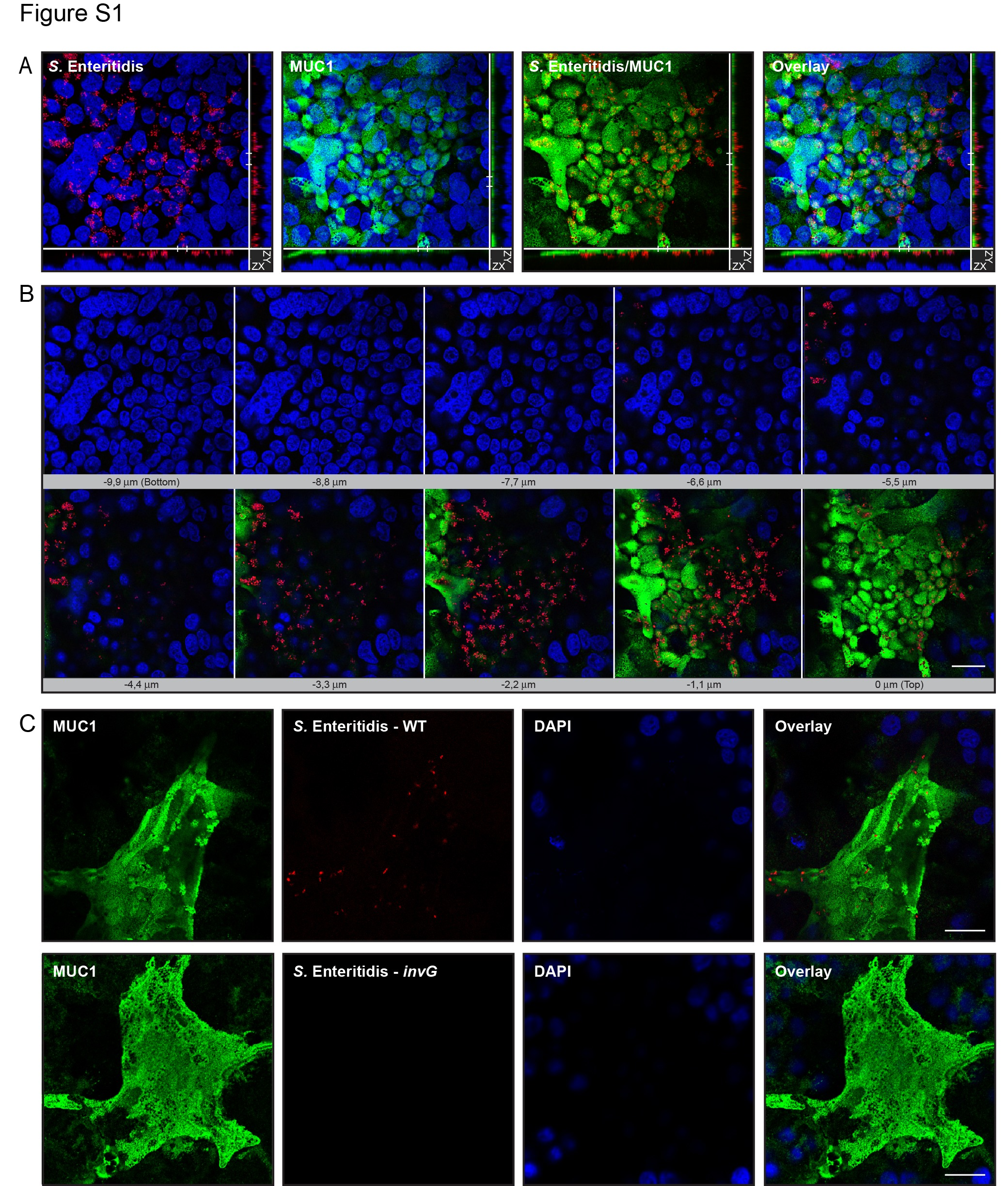

Supplement: S1 Fig — (A) Single channel maximum projections with orthogonal view of the image depicted in Fig 1B and 1C. MUC1: green, Salmonella: red, nuclei: blue. (B) Montage of different Z planes of the image depicted in Fig 1B and 1C. MUC1: green, Salmonella: red, nuclei: blue. (C) Immunofluorescence confocal microscopy imaging of confluent HT29-MTX cells infected with S. Enteritidis CVI-1 wild type or invG knockout bacteria (mCherry, red) stained for MUC1 (214D4, green) and nuclei (DAPI, blue). White scale bars represent 20 μm. (TIF) [file ppat.1007566.s001.tif]

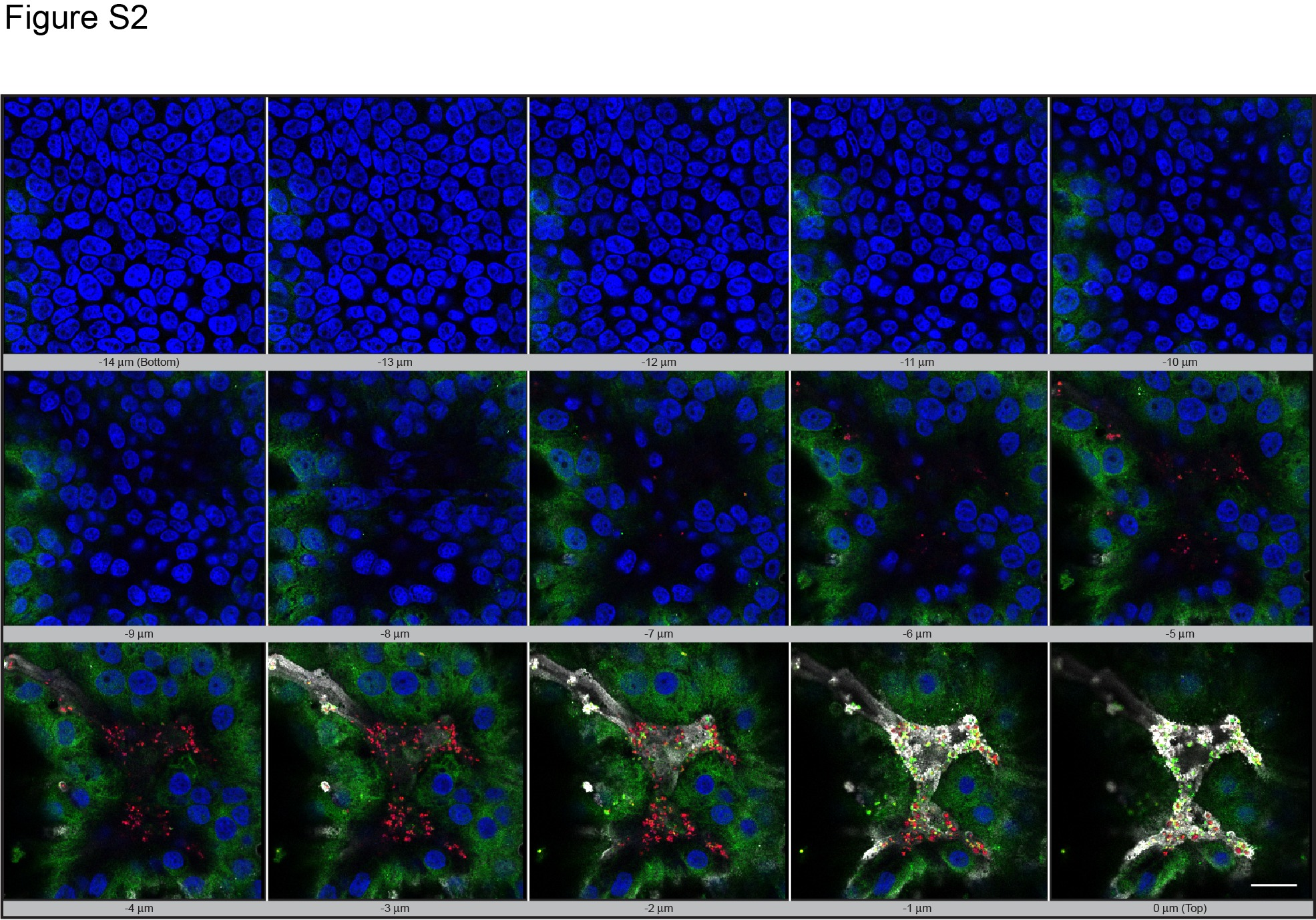

Supplement: S2 Fig — Montage of different Z planes of the image depicted in Fig 6A. MUC1: white, SiiE: green, Salmonella: red, nuclei: blue. (TIF) [file ppat.1007566.s002.tif]
